# Health-related behaviors and symptoms of anxiety and depression in Spanish nursing students: an observational study

**DOI:** 10.3389/fpubh.2023.1265775

**Published:** 2023-12-21

**Authors:** Enrique Ramón-Arbués, Lucía Sagarra-Romero, Emmanuel Echániz-Serrano, José Manuel Granada-López, Ana Cobos-Rincón, Raúl Juárez-Vela, Noelia Navas-Echazarreta, Isabel Antón-Solanas

**Affiliations:** ^1^Universidad San Jorge, Faculty of Health Sciences, Zaragoza, Spain; ^2^SAPIENF Research Group, University of Zaragoza, Zaragoza, Spain; ^3^GAIAS Research Group, University San Jorge, Zaragoza, Spain; ^4^Department of Physiatry and Nursing, University of Zaragoza, Faculty of Health Sciences, Zaragoza, Spain; ^5^Department of Nursing, Faculty of Health Sciences, University of La Rioja, Logroño, Spain; ^6^Biomedical Research Center of La Rioja, CIBIR, Logroño, Spain

**Keywords:** nursing students, mental health, anxiety, depression, health-related behaviors

## Abstract

**Background:**

Symptoms of anxiety and depression are prevalent among young adults and are a significant public health issue. College students are at a higher risk of experiencing poor mental health than other young people due to several factors, including moving away from home, financial pressures, heavy workload, poor time management skills, competitivity, new processes of socialization and insufficient coping mechanisms, among others. In addition, nursing students’ mental and physical health may also be affected by unhealthy lifestyle habits and health-related behaviors.

**Aim:**

To determine the prevalence of symptoms of depression and anxiety, and the association between these symptoms and health-related behaviors, in a population of Spanish student nurses.

**Methods:**

Cross-sectional study on a sample of 339 nursing students. We used the Hospital Anxiety and Depression Scale, the International Physical Activity Questionnaire-Short form, the Spanish Healthy Eating Index and an “*ad hoc*” questionnaire for sociodemographic variables. The association between psychological symptoms and health-related behaviors was analyzed through binary logistic regression models.

**Results:**

The prevalence of symptoms of depression and anxiety was 3.8% and 24.5%, respectively. Negative health-related behaviors were frequent, namely tobacco and alcohol consumption, suboptimal physical activity and diet. Moderate physical activity was associated with a lower probability of experiencing depressive symptoms. Symptoms of anxiety were related to a low level of physical activity, an unhealthy diet and alcohol consumption ≥2 times a week.

**Conclusion:**

Student nurses could benefit from the implementation of both physical and mental health promotion initiatives.

## Introduction

Symptoms of anxiety and depression are prevalent among young-adults, and are a significant public health issue that can have serious consequences in the short and long-term (1). Specifically, college students are at a higher risk of experiencing poor mental health than other people in the same age group (2). In fact, recent studies have reported high levels of psychological distress in this population. According to Franzoi et al. (3), 44.1% of students had anxiety and 20.4% experienced moderate to severe depression. In a cross sectional study of symptoms of depression in a large sample of first year students from Europe, Asia, Western Pacific, Latin-America and North America, Backhaus et al. (4) found that 48.1% of students had clinically significant symptoms of depression, ranging from 24 to 86% depending on the study location. In Spain, evidence on this topic is not abundant in the pre-COVID era. Two studies have reported symptoms of anxiety and depression in 23 and 18% of the university student population, respectively (5, 6). In recent years, scientific production on the subject has increased in order to determine the effects of the pandemic (confinement and other social distancing measures) on the mental health of Spanish college students. Méndez-Pinto et al. (7) detected symptoms of anxiety and depression in 39.8 and 15.5%, respectively. Similar figures, although slightly lower, were reported by Mosteiro-Diaz et al. (8) (31.6% anxiety symptoms and 11.4% depression symptoms). The truth is that this study, although necessary, probably does not reflect the reality of Spanish students nowadays; social distancing and other specific measures have gradually been withdrawn and, therefore it is necessary to create a new body of knowledge adapted to this new reality.

Several factors may influence college students’ mental health, namely moving away from home, financial pressures, heavy workload, poor time management skills, competitivity, new processes of socialization and insufficient coping mechanisms (9–11). Furthermore, healthcare students generally, and nursing students specifically, are exposed to other, potentially stressful, risk factors. For example, clinical placements require close human contact and a high level of emotional commitment; similarly, coping with sickness and death can result in higher levels of stress and fatigue (12). These are critical aspects that affect not only students’ mental health, but also both academic (13) and clinical (14) performance.

In addition to the above, nursing students’ mental and physical health may also be affected by unhealthy lifestyle habits and health-related behaviors. Previous studies have reported on the prevalence of unhealthy behaviors in nursing students. Heidke et al. (15) observed that 30.3% student nurses had a low level of physical activity, 10.5% were smokers, 17.4% consumed alcohol, and only 21.1% followed a healthy diet. Similarly, Macedo et al. (16) reported prevalences of 8.1%, 23.1%, 34.3%, and 80.8% of smoking, regular alcohol consumption, low physical activity, and insufficient intake of fruit and vegetables, respectively. In Spain, in a study involving 264 student nurses, Rodríguez-Muñoz et al. (17) reported that 11% of the students consumed alcohol twice per week, 15.5% smoked, 68.8% had a low level of physical activity and 70.5% followed an unhealthy diet or a diet in need of modifications.

The evidence available suggests that there is a relationship between unhealthy lifestyle habits and psychological symptoms in nursing students. For example, sedentary behaviors and unbalanced diets have been associated with depressive and anxiety symptoms, respectively (18, 19). In turn, Anosike et al. (20) reported an association between alcohol drinking and smoking, and anxiety and depression. Thus, student nurses could be at risk of experiencing poor mental health and wellbeing, not only due to external influences, but also due to a lack of adherence to healthy behaviors. High levels of psychological distress, when associated to unhealthy behaviors, may have a negative impact on their health and wellbeing and, thus, hinder the performance of the next nursing workforce generation. In Spain, this body of knowledge has not yet been developed and, to date, the nature and strength of the association between mental health and health-related behaviors among student nurses has not yet been clarified. For these reasons, we aimed to determine the prevalence of symptoms of depression and anxiety among Spanish student nurses, and to evaluate the association between depression and anxiety symptoms and health-related behaviors in this population. We hypothesized that student nurses who experienced psychological distress did not follow, or followed to a lower degree, a healthy lifestyle.

## Methods

### Design

We carried out a cross-sectional, descriptive study. Target population were student nurses registered in the Bachelor of Nursing program taught at the Faculty of Health Sciences of the San Jorge University (Spain). The tool Strengthening the Reporting of Observational Studies in Epidemiology (STROBE) was used to guide the reporting of our findings.

### Sample

Students were informed of the objectives of the research in their respective classrooms and during their class hours. In this way, all enrolled students were offered to participate, except those who for any reason did not attend class on the day assigned for recruitment in their classroom. Data collection for each participant was then carried out by completing a questionnaire. Due to the sensitive nature of the data, confidentiality and anonymity are ensured.

Participant recruitment and data collection took place in the classroom from February to May 2022. It was necessary to dedicate 4 months because our students alternate bimonthly periods of theoretical classes and clinical placements.

A total of 485 out of 682 student nurses were approached to take part in this investigation; the rest were unavailable during the period of recruitment and data collection. 120 out of 485 student nurses declined to participate (participation rate of 75.2% and refusal rate of 24.8%). A total of 365 responses were received, but 26 were excluded as they were either incomplete or invalid (data were glaringly untrue). Thus, the final sample of participants represented 49.7% of all enrolled students.

Sample size for the analysis of the association between health-related behaviors and psychological symptoms was calculated based on the hypothesis that student nurses with a low level of physical activity experience more depressive symptoms (61.3%) than those who are more active (41.4%) (21). Accordingly, for a potency of 90% and a α = 0.05, a sample size of 262 participants was needed (Epidat 4.2, SERGAS, Spain).

### Data collection

The data collection questionnaire comprised three sections: (1) sociodemographic variables, (2) health-related behaviors, and (3) psychological health. Information was collected about the participants’ age (<20; 20–24; ≥25 years), gender (male; female), living arrangement (alone; with mates; with family), weight and height (body mass index (BMI) = Kg/m^2^), average grade point (<7 points; 7–10 points), employment status (employed; unemployed), tobacco use (yes; no), screen-time (<4 h/day; 4–6 h/day; >6 h/day), and alcohol consumption (never or occasionally; at least once a week). Body mass index (BMI) was categorized as: <18.5 kg/m^2^ (underweight), 18.5–24.9 kg/m^2^ (normal weight), 25–29.9 kg/m^2^ (overweight) and ≥ 30.0 kg/m^2^ (obesity). In addition, validated questionnaires were used to assess physical activity, diet quality and symptoms of anxiety and depression.

Physical activity was evaluated through the International Physical Activity Questionnaire-Short form (IPAQ-SF). This tool assesses frequency, intensity, and duration of physical activity in the previous 7 days. Subsequently, participants were categorized based on their level of physical activity (22), namely low, moderate and high level of physical activity. The IPAQ-SF has been validated in the Spanish student college population and possesses adequate clinimetric properties (23).

Participants’ diet quality was evaluated through the Spanish Healthy Eating Index (SHEI) (24). The SHEI was adapted from Kennedy et al. (25) Healthy Eating Index and adapted to the Spanish context following the recommendations of the Spanish Society of Community Nutrition. This questionnaire comprises 10 items, scored from 0 to 10. Total score ranges from 0 to 100 and categorizes participants’ diet as healthy (>80 points), in need of modifications (50–80 points) and inadequate (<50 points).

Symptoms of depression and anxiety were evaluated using the Hospital Anxiety and Depression Scale (HADS). Originally developed by Zigmond and Snaith (26), this tool comprises 14 questions, 7 measuring symptoms of anxiety and 7 measuring symptoms of depression. All 14 items are measured on a 0–3 scale, with total scores ranging from 0 to 21 for each subscale. Three ranges were defined for each subscale: 0–7 (non-cases), 8–10 (doubtful cases) and 11–21 (cases). These cut-offs (8+ and 11+) were defined based on psychiatric ratings of anxiety and depression disorders (27). HADS is a tool initially designed for administration in the hospitalized population. Although its use has become popular among other populations in recent decades. Thus, Bjelland et al. (28) demonstrated that it works good for assessing the severity of anxiety and depression symptoms in all types of populations, including non-hospitalized populations. In particular, it is a tool used repeatedly in the study of university populations. The Spanish version of HADS has shown good internal consistency and external validity, with favorable sensitivity and specificity in the identification of cases of psychiatric disorder (29).

### Data analysis

Results from the descriptive analysis are presented as number and percentage. In addition, different binary logistic regression models (enter method) were employed to test the association between health-related behaviors and the probability of experiencing anxious and depressive symptomatology. Nagelkerke *R*^2^ was calculated to assess goodness of fit of the multivariate models. Data codification, processing and analysis were completed using the statistical software Statistical Package for the Social Science (SPSS version 21 for Windows, IBM Corp., Chicago, IL, United States) accepting a level of significance of *p* < 0.05.

### Ethical considerations

The research protocol was evaluated and approved by the Clinical Research Ethics Committee of Aragón before the start of this investigation (PI19/98). In addition, the co-authors confirm that national and international standards and regulations for ethical investigation with human subjects were observed and respected. Potential participants were informed about the study aims and methods of data collection by a member of the research team. Further, the student nurses were assured that participation was voluntary and that there would be no negative consequences to declining participating in this study. Consent to participate was implied by the completion and submission of the questionnaire.

## Results

Our participants were mostly women (83.8%), aged <20 (45.1%), had a normal weight (75.5%), lived with their families (60.5%), and were unemployed (77.6%) (Table 1).

**Table 1:** Sociodemographic characteristics of the participants.

Characteristics	*n* (%)
Gender	
Men	55 (16.2%)
Women	284 (83.8%)
Age (years)	
<20	153 (45.1%)
20–24	134 (39.5%)
≥25	52 (15.4%)
BMI categories	
Underweight	24 (7.1%)
Normal weight	256 (75.5%)
Overweight	57 (16.8%)
Obesity	2 (0.6%)
Living arrangement	
Alone	24 (7.1%)
With friends	110 (32.4%)
With family	205 (60.5%)
Employment status	
Unemployed	263 (77.6%)
Employed	76 (22.4%)
Average grade point (max. 10)	
<7 points	113 (33.3%)
7–10 points	226 (66.7%)

Prevalence of negative health-related behaviors was 29.2% for smokers and 47.2% for regular alcohol consumers. Also, 44.8% of subjects had low physical activity and up to 80.8% followed a diet in need of modifications. 80.5% of the participants spent 4 or more hours in front of a screen, excluding time spent working or studying. In terms of gender, statistically significant differences were observed in the prevalence of physical activity (more frequent in women) and alcohol consumption (more frequent in men). Prevalence of anxiety and depression symptoms were 24.5% and 3.8%, respectively, in our sample. In both cases, being a woman was associated with a higher probability of experiencing psychological symptoms (*p* < 0,05) (Table 2).

**Table 2:** Psychological health indicators and engagement in health behaviors of the sample.

	Total (*n* = 339)
**Psychological health indicators**	
Depression status	
No depression	297 (87.6%)
Borderline case	29 (8.6%)
Depressive symptomatology	13 (3.8%)
Anxiety status	
No anxiety	158 (46.6%)
Borderline case	98 (28.9%)
Anxious symptomatology	83 (24.5%)
**Health behaviors**	
Physical activity level	
Low	152 (44.8%)
Medium	108 (31.9%)
High	79 (23.3%)
Diet quality	
Inadequate	134 (39.5%)
Needs changes	140 (41.3%)
Healthy	65 (19.2%)
Screen-time (no studying/no working)	
<4 h/day	66 (19.5%)
4–6 h/day	170 (50.1%)
>6 h/day	103 (30.4%)
Smoking	
No	240 (70.8%)
Yes	99 (29.2%)
Alcohol consumption	
Never or occasionally	179 (52.8%)
Once a week	99 (29.2%)
≥2 times a week	61 (18.0%)

**Figure 1:**
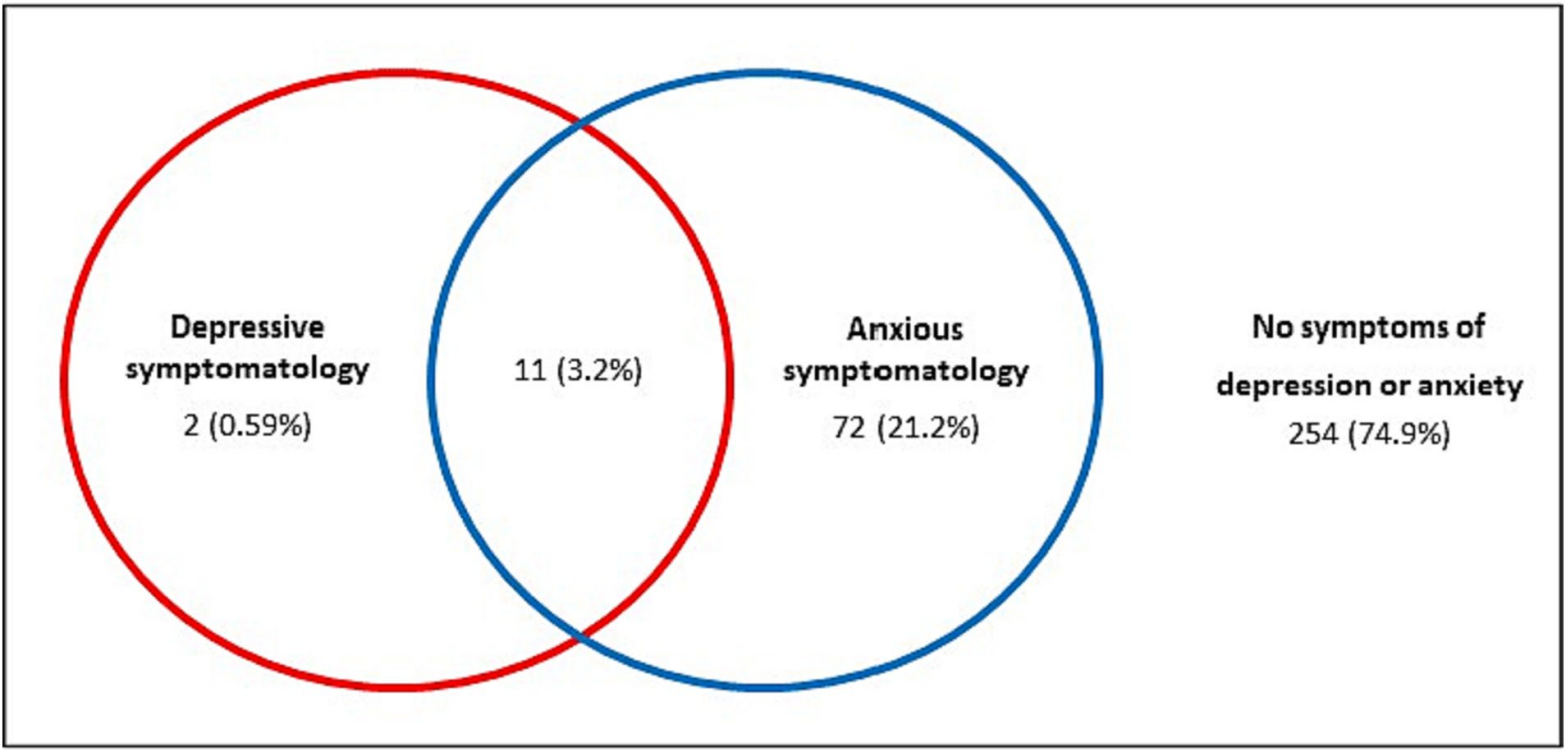
Coexistence of symptoms of depression and anxiety according to the results from HADS.

In addition, 3.2% of participants presented both symptoms of anxiety and symptoms of depression (Figure 1).

Binary logistic regression adjusted models suggested that physical activity was inversely related to symptoms of depression (aOR = 0.09 [CI95% 0.01, 0.80] for moderate physical activity) and to symptoms of anxiety (aOR = 0.36 [CI95% 0.19, 0.71] for moderate physical activity and aOR = 0.33 [CI95% 0.15, 0.71] for high physical activity). Symptoms of anxiety were predicted by following a diet in need of modifications (aOR = 4.90 [CI95% 1.61, 14.93]) or an inadequate diet (aOR = 7.06 [CI95% 2.33, 21.42]), and by regular alcohol consumption (≥2 times a week) (aOR = 4.24 [CI95% 1.64, 10.93]) (Tables 3, 4). The predictive capacity of the logistic regression models was 52.9% (Nagelkerke *R*^2^ = 0.529) for depressive symptoms and 35.1% (Nagelkerke *R*^2^ = 0.351) for symptoms of anxiety.

**Table 3:** Associations of health behaviors with depressive symptomatology (HADS-D score ≥ 11).

Health behaviors	OR	CI95%	Adjusted OR**	CI95%
Physical activity level				
Low (Ref.)	1		1	
Medium	0.12	(0.01, 0.94)*	0.09	(0.01, 0.80)*
High	0.16	(0.02, 1.29)	0.15	(0.02, 1.41)
Diet quality				
Healthy (Ref.)	1		1	
Needs changes	4.39	(0.54, 35.46)	2.59	(0.29, 23.02)
Inadequate	1.46	(0.14, 14.37)	1.38	(0.14, 13.57)
Screen-time (no studying/working)				
<4 h/day (Ref.)	1		1	
4–6 h/day	0.50	(0.11, 2.32)	0.43	(0.08, 2.24)
>6 h/day	1.29	(0.31, 5.38)	1.36	(0.30, 6.21)
Smoking				
No (Ref.)	1		1	
Yes	2.96	(0.97, 9.06)	1.29	(0.37, 4.49)
Alcohol consumption				
Never or occasionally (Ref.)	1		1	
Once a week	0.76	(0.19, 3.03)	1.58	(0.38, 6.61)
≥2 times a week	1.27	(0.31, 5.07)	3.02	(0.56, 16.10)

^*^p < 0.05. ^**^Adjusted for sex, age, BMI, living arrangement, employment status and average grade point.

**Table 4:** Associations of health behaviors with anxious symptomatology (HADS-A score ≥ 11).

Health behaviors	OR	CI95%	Adjusted OR**	CI95%
Physical activity level				
Low (Ref.)	1		1	
Medium	0.38	(0.21, 0.70)*	0.36	(0.19, 0.71)*
High	0.38	(0.19, 0.75)*	0.33	(0.15, 0.71)*
Diet quality				
Healthy (Ref.)	1		1	
Need changes	5.08	(1,72, 14,99)*	4.90	(1.61, 14.93)*
Inadequate	7.45	(2,54, 21,82)*	7.06	(2.33, 21.42)*
Screen-time (no studying/working)				
<4 h/day (Ref.)	1		1	
4–6 h/day	0.78	(0.37, 1.61)	0.86	(0.41, 1.78)
>6 h/day	1.00	(0.52, 1.92)	0.74	(0.33, 1.66)
Smoking				
No (Ref.)	1		1	
Yes	1.77	(1.05, 2.98)*	1.63	(0.88, 3.00)
Alcohol consumption				
Never or occasionally (Ref.)	1		1	
Once a week	1.35	(0.78, 2.33)	1.01	(0.55, 1.83)
≥2 times a week	2.59	(1.10, 6.10)*	4.24	(1.64, 10.93)*

^*^p < 0.05. ^**^Adjusted for sex, age, BMI, living arrangement, employment status and average grade point.

## Discussion

The aim of this investigation was to examine the prevalence of symptoms of depression and anxiety in a sample of Spanish student nurses, and to analyze the association between these symptoms and health-related behaviors.

Psychological distress among student nurses has been analyzed before in different contexts. For instance, in a systematic review and metanalysis of 27 cross-sectional studies and a global sample of 8,918 nursing students, Tung et al. (14) identified a prevalence of depression of 24%. Chernomas and Shapiro (30) detected symptoms of depression and anxiety in 32 and 29% of Canadian nursing students, respectively. Recently, similar studies carried out in different contexts have reported variable rates of symptoms of depression and anxiety, namely Turkey (signs of depression: 55.5%, signs of anxiety: 50.9%) (31), Saudi Arabia (signs of depression: 43.3%, signs of anxiety: 37.2%) (32), Japan (signs of depression: 18.3%, signs of anxiety: 34.6%) (33), and Brazil (signs of depression: 54.2%, signs of anxiety: 40.1%) (34). A recent metanalysis (35) of 64 studies and a pooled sample of 100,187 college students identified a prevalence of symptoms of depression and anxiety of 33.6 and 39.0%, respectively. Our findings substantially differ from these results, with approximately a quarter of the student nurses who participated in our study presenting signs of anxiety (24.5%) and only a minority evidencing signs of depression (3.8%). These findings contradict the theory that health sciences students present higher levels of anxiety and depression than other students due to being exposed to a higher number of stressors (36, 37). This may be explained by the fact that socioeconomic contexts and tools used in our study and the studies integrated in the metanalysis were heterogenous and, therefore, not directly comparable. In addition, there were significant differences in the timing of previous investigations and that of our study. Whilst most of the studies around this topic were carried out during the COVID-19 pandemic, ours was developed at the beginning of 2022. After two years of restrictions limiting social contact, the year 2022 was marked by a certain degree of post-pandemic euphoria following the withdrawal of all restrictive measures in Spain. Research around this phenomenon is still scarce, although many agree that, during the pandemic, we all underwent a traumatic period of psychological distress (38), followed by a wave of positivity as the COVID-19 pandemic eased and people ventured out again into the world (39).

We identified a high prevalence of negative health-related behaviors in our sample, including a low physical activity level (44.8%), inadequate diet quality (39.5%), prolonged screen-time (30.4%), smoking (29.2%) and regular alcohol consumption (18%). Our results are similar to those obtained in previous studies in the Spanish college context (6, 40, 41). This implies that there is a good chance that these unhealthy behaviors have a negative impact not only on the student nurses’ health and wellbeing in the medium to long term, but also on their professional performance, as the evidence suggests that lifestyle habits of healthcare professionals influence their health promotion practice (42, 43).

It is important to highlight that negative health-related behaviors were associated with psychological distress. Regular physical activity was inversely associated with symptoms of anxiety and depression. This association which, from a neurobiological point of view, is based on the activation of the endocannabinoid system and the up-regulation of the brain-derived neurotrophic factor (44), has been reported in previous studies in the general population (45) and in young adults (46, 47). Similarly, previous studies have reported significant associations between healthy dietary habits (caloric restriction, breakfast consumption or no snacking) and specific foods and nutrients intake (fruits and vegetables, omega-3 fatty acids or Zinc), and good mental health (48, 49). Accordingly, following a healthy diet was strongly, inversely associated with experiencing symptoms of anxiety in our sample.

Regular alcohol consumption (≥2 times a week) was potently associated with anxiety states. Kushner et al. (50) examined the relative, prospective risk of alcohol dependency and several common diagnoses of anxiety in approximately 500 college students. Although anxiety disorders were more frequent than alcohol dependency, the prospective risk of experiencing either of these disorders in a follow-up assessment was two to five times higher if the other condition was present in a previous evaluation. That is, both conditions substantially increased the prospective, relative risk of developing the other, in what looks like a bidirectional relationship. From the point of view of psychological theory and according to Tension Reduction Theory, the primary motive for drinking alcohol for some people is coping with negative emotions (51, 52). On the other hand, from a neurobiological point of view, it is known that the first stages of alcohol addiction (binge drinking/intoxication) activate reward systems (for example, secretion of dopamine and opioid peptides in the ventral striatum) (53). Thus, positive reinforcement of this activation motivates continuous and increasing alcohol use.

Although the level of psychological distress in our sample was lower than that reported in previous studies, the prevalence of anxiety amongst student nurses was relatively high. Thus, attention should be paid by all the relevant stakeholders, including health policymakers and higher education authorities and decision-makers, to college students’ mental health, health-related behaviors, and lifestyle. Most, if not all, higher education institutions offer counsel and support to students through a myriad of different services, such as student orientation and counselling services, and mentorship programs (54, 55). However, they are rarely tailored to the specific needs of students from particular, traditionally more stressful disciplines, such as nursing, nor are their interventions evidence-based (18). For example, student nurses could benefit from specific strategies addressing degree and/or profession-specific stressors, such as clinical simulation. Clinical placements are often described as stressful and a source of anxiety by student nurses; integrating clinical simulation scenarios into the nursing curriculum may be helpful to develop the students’ self-confidence and coping mechanisms when dealing with emotionally complex situations in the clinical setting (56, 57). Different strategies have been proposed to address this issue, including behavioral interventions (58, 59), and mindfulness (60, 61). However, the quality of these investigations was variable and, generally, samples were small and there was no, or limited, follow-up.

To our knowledge, this is the first study analyzing the association between psychological distress and health-related behaviors in student nurses in our context. Our results underline the need to periodically assess the psychological symptoms and health habits of nursing students to implement early measures. This study has several strengths, which add to the validity and representativeness of our findings, namely standardized methods of data collection were used, both psychological distress symptoms and health-related behaviors were analyzed using validated tools, and the associations established between variables are plausible.

However, we would like to highlight some limitations to our investigation. Our sample was recruited from one single higher education institution, which may affect the generalizability of our findings to the general population of Spanish student nurses. Having said this, the sociodemographic characteristics of our sample coincide with those of the nursing students enrolled in most nursing degree programs across our nation. The study design allows us to establish associations between variables, but it does not allow us to establish causal relationships between them. Future longitudinal studies should analyze the associations established in this investigation, especially how mental health issues affect health-related behaviors in this population, and vice versa. Data collection was carried out during the months of February to May. In our context, the final exams are proposed in the month of June, therefore, it cannot be ruled out that those recruited in May may be subject to higher levels of stress and anxiety. In this study, the academic year, a factor that could potentially be associated with unhealthy lifestyle habits, anxiety, and depression in nursing students, was not included in the analysis. This was a voluntary decision by the research team based on two fundamental issues. Firstly, in our environment a considerable number of students repeat subjects or are partially enrolled in nursing studies, so it is common to find students with 3 or 4 years of permanence in the University taking subjects from the 1st academic year. Secondly, Spanish educational legislation allows the validation of subjects based on previous studies, not necessarily from Higher Education, by the students. Thus, it is common to find students in their 1st year of access to Nursing studies, taking subjects from the 3rd or 4th academic year. For these reasons, the academic year variable could be, in our setting, more of a confounding factor than an adequate predictor of unhealthy lifestyle habits, depression and anxiety.

## Conclusion

The results from this investigation evidence a considerable prevalence of symptoms of psychological distress in the population of Spanish student nurses. In addition, there is a clear association between symptoms of anxiety and unhealthy behaviors. This suggests that student nurses are at risk of experiencing negative health outcomes in the medium to long term. Also, their future professional performance as qualified nurses may be hindered by long term exposition to psychological distress and burnout (62). These findings underline the need to implement both physical and mental health promoting strategies in the higher education context. Including behavioral interventions, mindfulness and mentorship programs in nursing undergraduate curricula may contribute to improving health-related behaviors in this population and equipping them with tools to increase resiliency and manage psychological distress.

## Data availability statement

The raw data supporting the conclusions of this article will be made available by the authors, without undue reservation.

## Ethics statement

The studies involving humans were approved by Comité de Ética de Investigación Clínica de Aragón. The studies were conducted in accordance with the local legislation and institutional requirements. The participants provided their written informed consent to participate in this study.

## Author contributions

ER-A: Conceptualization, Formal analysis, Project administration, Writing – original draft. LS-R: Investigation, Writing – original draft, Project administration, Validation. EE-S: Investigation, Data curation, Resources, Writing – original draft. JG-L: Conceptualization, Data curation, Writing – review & editing, Formal analysis. AC-R: Writing – review & editing, Investigation, Software, Visualization. RJ-V: Conceptualization, Data curation, Funding acquisition, Writing – review & editing. NN-E: Formal analysis, Methodology, Validation, Writing – review & editing. IA-S: Supervision, Validation, Visualization, Writing – original draft.
